# Head and Neck Reconstruction

**Published:** 2013-02-18

**Authors:** Adam M. Feintisch, Ramazi Datiashvili

**Affiliations:** Department of Surgery, Division of Plastic Surgery, New Jersey Medical School, University of Medicine and Dentistry of New Jersey, Newark

## DESCRIPTION

A 22-year-old female presented with an acquired mandibular defect status post radical resection of mandibular osteosarcoma. Patient subsequently underwent fibular osteocutaneous free flap reconstruction.

## QUESTIONS

1. **What anatomical and functional considerations must be taken into account when reconstructing defects of the upper aerodigestive tract?**
2. **What methods of reconstruction are available after head and neck tumor extirpation?**
3. **How are defects of the floor of mouth, tongue, buccal cavity, and hypopharynx typically reconstructed?**

## DISCUSSION

Because of the complex functions and anatomy of the upper aerodigestive tract, reconstruction becomes a significant challenge. Reconstruction must take into account not only the esthetic concerns of the patient but also the functionality of the oral cavity, oropharynx, and hypopharynx including speech, mastication, bolus preparation and manipulation, deglutition, oral and oronasal competence, and early digestion.^1^^,^^2^^,^^5^ As many head and neck tumor extirpation procedures concomitantly involve neck dissections, reconstructive goals must also include separation of the upper aerodigestive tract from involved vital neck structures to avoid complications such as salivary leak and orocutaneous fistulas.^1^^,^^2^

Head and neck reconstruction follows the principles of the reconstructive ladder. Primary closure can accomplish a fast and uncomplicated reconstruction when used for small defects. With moderate-sized defects, local flaps may be used, replacing resected tissue with like tissue and affording quality match in texture, color and bulk. Previously irradiated tissue may limit the usefulness of local flaps as well as skin grafts.^1^^,^^2^ Skin grafts are typically used when only skin or mucosal lining is needed.^1^ For larger defects of the oral cavity, pedicled regional myocutaneous flaps may reconstruct defects without the added complexity of microsurgical procedures. Flaps include the deltopectoral, pectoralis major, forehead, latissimus, and trapezius flaps. They do, however, have the added morbidity of donor-site deformities, unappealing esthetic outcomes, and, at times, questionable vascular perfusion.^1^^-^3 This is particularly concerning at a flaps' most distal point, a site where the integrity of reconstruction is most crucial. When defects are too remote, large, or complex for a pedicled reconstruction, free tissue transfer may be more practical. This allows for composite reconstruction of defects involving multiple tissue types including skin, mucosa, muscle, and bone. When appropriate microsurgical techniques are employed, free flaps afford the ability to bring a reliable and superior blood supply to the acquired defect.^1^^,^^2^ They have the added benefit of a lower complication rate, albeit a higher morbidity when complications do arise, such as partial or total flap loss. Mucosal defects may be reconstructed using a radial forearm free flap, while those defects involving both mucosa and bone typically employ the use of a free fibula flap. Composite scapular, iliac, and radial forearm flaps may be used; however, these are utilized less frequently.^1^^,^^2^

Reconstruction of the floor of the mouth (FOM) requires tissue that is soft, provides adequate free tongue mobility, and, if possible, is sensate.^1^^-^3 Although small defects may be resurfaced with skin grafts, scarring, contracture, and tongue tethering may be problematic.^2^ Regional flaps (ie, nasolabial, forehead, facial artery musculomucosal, and deltopectoral) are mainly historical as disadvantages superseded benefits with improvements in microsurgery. Typically, such flaps had questionable or tenuous vascular perfusion, donor-site defects, and were precluded from use in irradiated areas.^2^ Some required creation of a temporary orocutaneous fistula and frequently a second operation for division and inset.^2^ Defects related to FOM are best reconstructed using free tissue transfer. Options include radial forearm, anterolateral thigh (ALT), and temporalis fascial flaps. The former is particularly useful owing to its thin and pliable nature, allowing for free movement of the tongue and food boluses.^1^^-^3 The majority of these flaps can be made sensate.^1^^-^3

Tongue reconstruction is particularly challenging due to its complex muscular anatomy and participation in all aspects of oral functioning. For defects leaving more than 20% to 30% of the tongue intact, reconstruction should focus on maintaining full mobility of the tongue remnant.^2^^,^^3^ This can be accomplished with the use of a thin and pliable flap, namely a radial forearm free flap. An ALT flap is another option. When less than 20% to 30% of the tongue remains, reconstructive efforts must focus on bulk rather than mobility, as residual function is frequently unavailable.^2^^,^^3^ Glossectomy reconstructions typically involve use of a rectus abdominis, ALT, or scapula/parascapular free flap. These flaps supply ample bulk that allows the neotongue to participate in deglutition and direct secretions laterally toward the oropharynx.^1^^-^3

Defects of the buccal cavity may be reconstructed in a similar fashion to FOM defects. Primary closure, skin grafts, or mucosal grafts may be used for small defects. A temporoparietal fascial flap may also be used by tunneling under the zygomatic arch and insetting with an overlying skin graft for intraoral lining.^2^ Because of its limitations in arc of rotation and size, additional flaps may need to be employed for larger defects including a pedicled pectoralis major flap or more commonly, a free radial forearm flap.^1^^-^3

When tumor extirpation leaves laryngeal, pharyngeal, or cervical esophagus defects, goals of reconstruction focus on restoring a patients' ability to speak, swallow without aspirating, and breath without a tracheotomy.^1^^,^^2^^,^^4^ The majority of resections, however, are not amenable to complete functional restoration and patients frequently require a permanent stoma to assist with breathing and various speech modalities. Instead, preserving quality of life tends to be the main determinant of reconstructive efforts given the low 5-year survival rates (25%-35%) seen in advanced cervical aerodigestive carcinoma.^2^^,^^5^ For pharyngoesophageal reconstruction, it is important to create a thin-walled conduit to allow for the passage of food boluses. Methods of reconstruction include primary closure, locoregional flaps (laryngeal, cervical, deltopectoral, pectoralis, and latissimus flaps), gastric “pull-up,” and colonic transpositions.^2^^,^^4^ With advancements in microsurgical techniques, jejunal and tubed radial forearm flaps are particularly useful for circumferential defects. The ALT flap has also gained popularity.^1^^,^^2^^,^^4^

Our patient is a 22-year-old woman with biopsy-proven high-grade osteosarcoma. The patient had extreme difficulty with both speech and mastication secondary to the size of her tumor. She underwent composite resection of the right mandible, floor of mouth, and selective neck dissection levels I to III. The patient then underwent reconstruction with an osteocutaneous fibula free flap and mandibular plating.

## Figures and Tables

**Figure 1:**
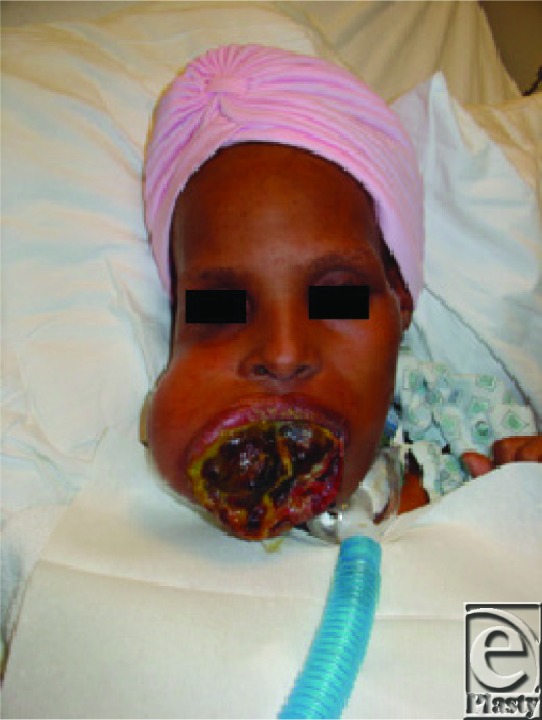
Preoperative photograph of mandibular osteosarcoma

**Figure 2:**
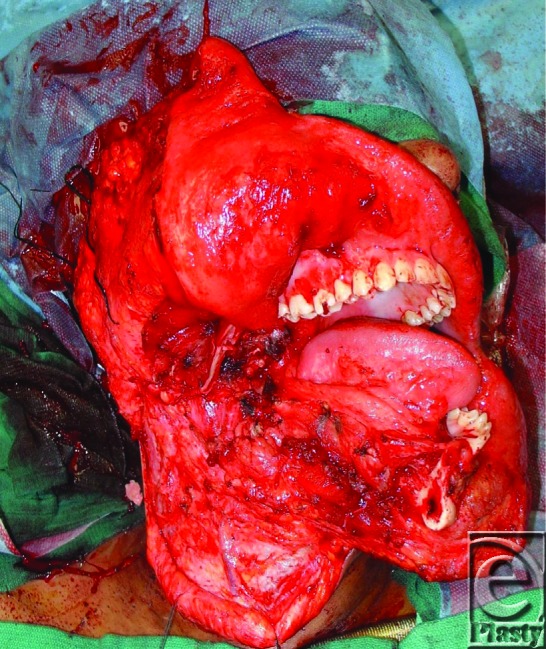
Intraoperative defect status post tumor extirpation.

**Figure 3:**
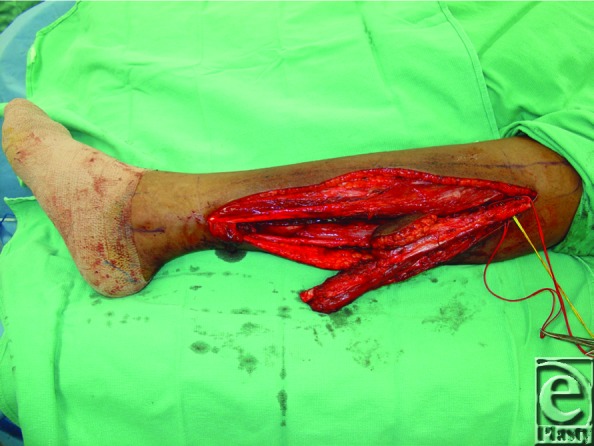
Raised fibular osteocutaneous free flap prior to division of peroneal vessels.

**Figure 4:**
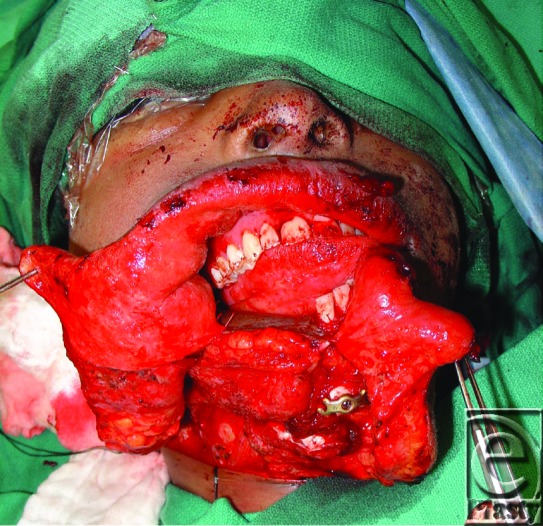
Flap inset with intraoral skin paddle lining floor of mouth. Mandibular reconstruction bar can be seen.

**Figure 5:**
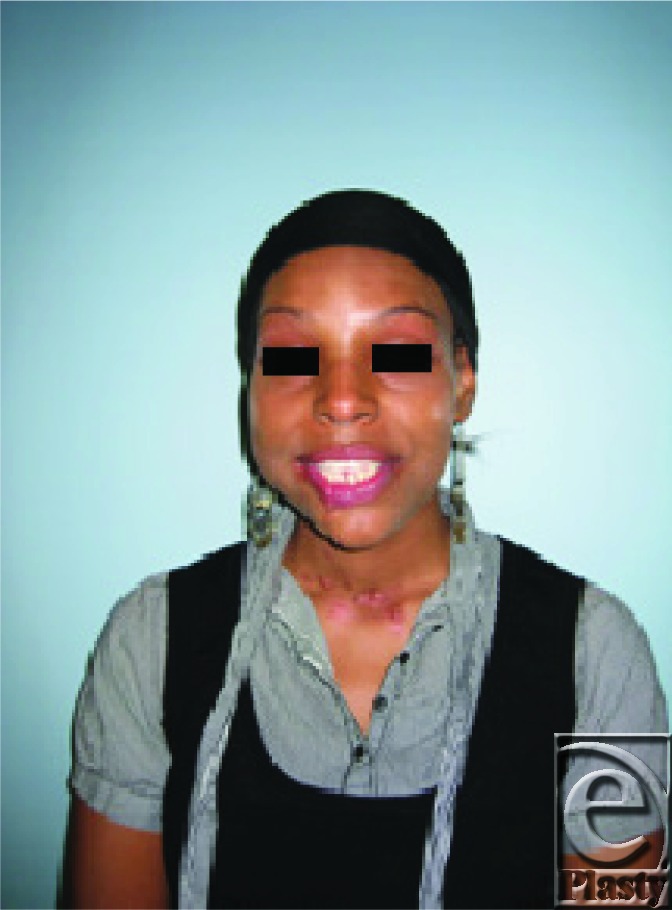
Final postoperative result at 7 months.
